# Supplementary figures and images for: Transcriptome sequencing reveals the evolutionary histories and gene expression evolution in two related Pagurus species
Source: PLoS One. 2025 Aug 20;20(8):e0330170. doi: 10.1371/journal.pone.0330170 (PMC12367144; doi:10.1371/journal.pone.0330170)

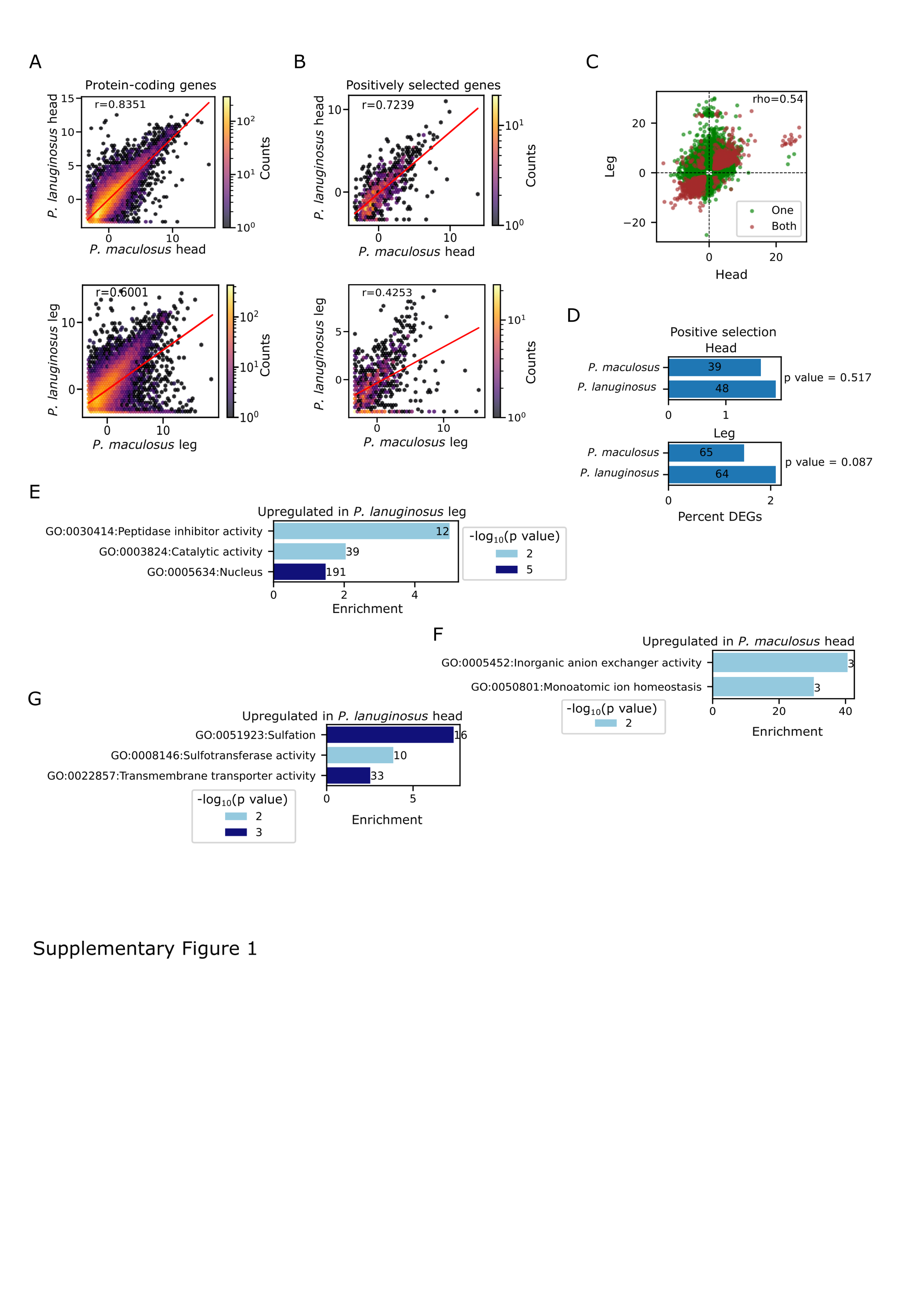

Supplement: S1 Fig — A. 2-D hexagonal binning plots showing the relationships between protein-coding gene expression patterns of the P. maculosus (PM) and P. lanuginosus (PL) in the head (upper panel) and the leg (lower panel). B. 2-D hexagonal binning plots showing the relationships between the positively selected protein-coding gene expression patterns of the P. maculosus (PM) and P. lanuginosus (PL) in the head (upper panel) and the leg (lower panel). For A and B, r = Pearson’s correlation coefficient while the red lines represent the linear regression lines. Log2-transfromed TMM-normalized expressions were used for the plots C. Scattered plot showing the relationship between the log-fold changes of genes differentially expressed in head and/or leg. Each dot represents a gene. Negative values represent P. lanuginosus enrichment while positive values represent P. maculosus enrichment. Rho = Spearman’r correlation coefficient. D. Species bias of positively selected differentially expressed head (upper) and leg (lower) genes. P values were computed using Fisher exact tests. Gene ontology enrichments of genes are upregulated in P. lanuginosus leg (E), P. maculosus head (F) and P. lanuginosus head (G). The bar color corresponded to the Bonferroni-corrected hypergeometry p value. (TIF) [file pone.0330170.s001.tif]
