# Supplementary material for: Transcriptome sequencing reveals the evolutionary histories and gene expression evolution in two related Pagurus species
Source: PLoS One. 2025 Aug 20;20(8):e0330170. doi: 10.1371/journal.pone.0330170 (PMC12367144; doi:10.1371/journal.pone.0330170)
Supplement: Attachment — Submitted filename: Response to Reviewers.docx [file pone.0330170.s008.docx]

We appreciate the editor and the reviewers for the time and efforts spent in reviewing our manuscript. We are particularly grateful for the suggestions towards the improvement of the manuscript. We have meticulously revised the manuscript based on the suggestions and comments from the editor and the reviewers. We have also corrected other typographical or grammatical errors that we encountered. All changes have been marked in one of the uuploaded documents. Specific response and changes made in response to each comment are itemized below (in blue font).

**Response to the editor**

We are grateful to the editor for the comments. We have responded to each raised comment with blue font below.

1. Please ensure that your manuscript meets PLOS ONE's style requirements, including those for file naming. The PLOS ONE style templates can be found at

https://journals.plos.org/plosone/s/file?id=wjVg/PLOSOne_formatting_sample_main_body.pdf and

[https://journals.plos.org/plosone/s/file?id=ba62/PLOSOne_formatting_sample_title_authors_affiliations.pdf](https://track.editorialmanager.com/CL0/https:%2F%2Fjournals.plos.org%2Fplosone%2Fs%2Ffile%3Fid=ba62%2FPLOSOne_formatting_sample_title_authors_affiliations.pdf/1/010f0197b0e0d229-5852670b-e93c-4992-b6a8-c66af2b35072-000000/nmBEjqU40mjg1Y-EwmEKUhSgpmCnttHQXoSKkURz3-0=217)

We appreciate the editor for pointing this out. The first page has been revised to be consistent with PLOS ONE’s style. We have also revised the listing of supplementary figures and tables to be consistent with the PLOS ONE’s style.

2.  In your Methods section, please provide additional information regarding the permits you obtained for the work. Please ensure you have included the full name of the authority that approved the field site access and, if no permits were required, a brief statement explaining why.

We have updated the Materials and Methods section by adding the following sentences. “Sample collection permit for experimental research with number 5-2 was granted by Wakayama Prefecture, Japan. Researches on Pagurus species do not require any special approval. All experiments complied with institutional regulations and Japanese policy on animal use [22].” We also corrected the sampling date to reflect the actual date when the specific sequenced samples were collected.

3. We note that the grant information you provided in the ‘Funding Information’ and ‘Financial Disclosure’ sections do not match.

When you resubmit, please ensure that you provide the correct grant numbers for the awards you received for your study in the ‘Funding Information’ section.

The grants used for the study do not have any special grant number as they are mostly in-house grants.

4. Thank you for stating the following financial disclosure:
“This study was supported by the funding of Research Organization of Information and Systems (ROIS) for special collaboration research student program of National Institute of Genetics (NIG) and Kyoto University, Japan”

Please state what role the funders took in the study. If the funders had no role, please state: "The funders had no role in study design, data collection and analysis, decision to publish, or preparation of the manuscript."
If this statement is not correct you must amend it as needed.
Please include this amended Role of Funder statement in your cover letter; we will change the online submission form on your behalf.

We declare that "The funders had no role in study design, data collection and analysis, decision to publish, or preparation of the manuscript."

5. Thank you for stating the following in the Acknowledgments Section of your manuscript:
“The authors are thankful to Mr. Nakano, R., Ms. Nakano, M., and the members of SMBL for their help in collecting the study animals. This study was supported by the funding of Research Organization of Information and Systems (ROIS) for special collaboration research student program of National Institute of Genetics (NIG) and Kyoto University, Japan.”

We note that you have provided funding information that is not currently declared in your Funding Statement. However, funding information should not appear in the Acknowledgments section or other areas of your manuscript. We will only publish funding information present in the Funding Statement section of the online submission form.
Please remove any funding-related text from the manuscript and let us know how you would like to update your Funding Statement. Currently, your Funding Statement reads as follows:
“This study was supported by the funding of Research Organization of Information and Systems (ROIS) for special collaboration research student program of National Institute of Genetics (NIG) and Kyoto University, Japan”

Please include your amended statements within your cover letter; we will change the online submission form on your behalf.

As suggested by the editor, we have deleted the funding-related text from the manuscript. And we would like to leave the funding statement as it is, that is, “This study was supported by the funding of Research Organization of Information and Systems (ROIS) for special collaboration research student program of National Institute of Genetics (NIG) and Kyoto University, Japan”

6. When completing the data availability statement of the submission form, you indicated that you will make your data available on acceptance. We strongly recommend all authors decide on a data sharing plan before acceptance, as the process can be lengthy and hold up publication timelines. Please note that, though access restrictions are acceptable now, your entire data will need to be made freely accessible if your manuscript is accepted for publication. This policy applies to all data except where public deposition would breach compliance with the protocol approved by your research ethics board. If you are unable to adhere to our open data policy, please kindly revise your statement to explain your reasoning and we will seek the editor's input on an exemption. Please be assured that, once you have provided your new statement, the assessment of your exemption will not hold up the peer review process.

We have made the raw read data publicly available (as from July 22nd, 2025) using the new accessions provided in the manuscript. The data can now be assessed from <https://www.ncbi.nlm.nih.gov/bioproject/PRJDB35803.> We would like to point out that we discovered that the biological replicates were not correctly entered in the original submission (<https://www.ncbi.nlm.nih.gov/bioproject/?term=PRJDB7354>). That is why we had to revise the submission and now have new accessions for the project and the samples. The manuscript has been appropriately revised to state “All raw reads generated in this study are available in the DDBJ Sequence Read Archive (DRA) under the BioProject accession number PRJDB35803. The data sets supporting the results presented here are available with the accession numbers DRR708008-DRR708019.”

7. PLOS requires an ORCID iD for the corresponding author in Editorial Manager on papers submitted after December 6th, 2016. Please ensure that you have an ORCID iD and that it is validated in Editorial Manager. To do this, go to ‘Update my Information’ (in the upper left-hand corner of the main menu), and click on the Fetch/Validate link next to the ORCID field. This will take you to the ORCID site and allow you to create a new iD or authenticate a pre-existing iD in Editorial Manager.

ORCID ID has been linked and validated by Editorial Manager.

8. We are unable to open your Supporting Information file [HermitCrab_SupplementaryTables.xlsx and S1Fig.eps]. Please kindly revise as necessary and re-upload.

We have confirmed that the files are still in good shape. We suspect that the problem might have arisen during file uploading. We will re-upload the files during the submission of the revised manuscript. To ensure that the files can be opened, supplementary figure will be saved in two formats (.eps and .tiff). An additional compressed file of the supplementary figure and table files will also be attached. Multiple uploading of the same files is to ensure the ability of the files to be opened.

9. Please review your reference list to ensure that it is complete and correct. If you have cited papers that have been retracted, please include the rationale for doing so in the manuscript text, or remove these references and replace them with relevant current references. Any changes to the reference list should be mentioned in the rebuttal letter that accompanies your revised manuscript. If you need to cite a retracted article, indicate the article’s retracted status in the References list and also include a citation and full reference for the retraction notice.

The revised manuscript now included references 18-20 supporting the growing use of molecular barcodes. We have listed reference 22 about institutional regulations and Japanese policy on animal use. In addition, we have cited new papers (references 55-58) suggested by a reviewer.

**Response to reviewers**

Reviewer #1: I believe that this paper is valuable for publication. However, as mentioned below, it needs some revisions.

We appreciate the reviewer for the time and efforts towards the improvement of our manuscripts. We are sincerely grateful for the multiple corrections that the reviewer has suggested. We have incorporated the reviewer’s suggestions and corrections in the revised version of the manuscript. We have also listed point-by-point response to the reviewer’s suggestion and comments.

L.38, 39: leg of xxx leg?

This has been revised by deleting “leg”.

L.40: chin-based?

“chin-based” has been changed to “chitin-based”

L.52: what is “those species”?

“those species” has been changed to “*Pagurus* species”

L.52: “Sibling species and cryptic species are sometimes not properly identified”. Because taxa not known are cryptic species.

This sentence has been changed to “Consequently, many sibling species remain cryptic species that are difficult to recognize using traditional classic methods”

L.93: “RNA samples were extracted separately from the head and leg” Both parts contain muscle, membrane, and exoskeleton or something else, but RNA was extracted from muscle. First, the material must be properly identified.

We apologize for this confusion. RNA extraction was actually done using the whole leg or head as pointed out in Figures 1A and B, and not just for the muscle. We have replaced “muscle” with “head or leg” in the Methods section.

BLAST not blast

All instances of “blast” to “BLAST”, including “BLASTN” and “BLASTP”.

L.211-212: This must be placed in Materials and Methods.

This has been moved.

L.215: remove “for each sample” How many individuals per species were used?

This has been revised. Three individuals were used per species.

L215-216: This must be placed in Materials and Methods.

This has been removed.

L.219-221: This must be placed in Materials and Methods.

This has been deleted. We have revised the following sentence to read “Removal of potentially noisy transcripts produced 203,793 transcripts, including 140,954 genes for *P. maculosus,* and 259,375 transcripts, including 179,685 genes for *P. lanuginosus* (**Table 1**)”

L.242-244: This must be placed in Materials and Methods.

This has been deleted. We have added the following to give the background to the result: “Transcriptome assembly has the potential to retrieve both coding and noncoding transcripts. We therefore checked the proportion of both coding and noncoding transcripts in our transcriptome assembly (see Materials and Methods)”

L.321-324: References 9 and 18 addressed only the genus Pagurus. Therefore, if the authors are referring to the non-monophyletic status of Pagurus in relation to the family Lithodidae, they should cite other references (e.g., Cunningham et al., 1992; Zaklin, 2001; Morrison et al., 2002; Blacken-Grissom et al., 2013).

We appreciate the reviewer. We have rephrased the sentence to focus on the evolution of *Paralithodes* species from *Pagurus* lineage. As suggested by the reviewer, we have added Cunningham *et al.* (1992), Morrison *et al.* (2002) and Bracken-Grissom *et al.* (2013), as suggested by the reviewer. We also added Chow et al. (2023) which supports the same hypothesis. The sentence now reads “This result further confirmed the previous studies reporting “hermit-to-king” hypothesis that *Paralithodes* species evolved from *Pagurus* lineage [55–58].”.

L.505: respectively

“respective” has been changed to “respectively”

The generic abbreviations for Pagurus, Paralithodes, and Petrolisthes should be clear. For example, they could be Pag., Par., and Pet.

We appreciate the reviewer for this suggestion. Since our focus is on *Pagurus*, we have retained *P.* for *Pagurus* genus while using *Par.* and *Pet.* for *Paralithodes*, and *Petrolisthes*, respectively, as suggested by the reviewer.

L.505-: I guess that almost all nucleotide substitutions in protein-coding mitochondrial DNA between the two Pagurus species are synonymous.

In Sultana *et al.*, two rRNAs, two ATPs, *Cox1*, *Cox2*, *Cox3*, *Nn2*, and *Nd3* mitochondrial regions were analyzed to compute overall nucleotide distance. Although the mitochondrial genes included those that code and those that do not code for proteins, all regions were analyzed together without considering nucleotide substitution type. Indeed, one of the motivations for this study was to more systematically investigate nucleotide evolution. In this study, we have separately computed synonymous and nonsynonymous substitution rates in larger gene set.

L.519: “found” should be “estimated”

This has been corrected.

L.525: specify the two species.

“the two species” has been changed to “*P. lanuginosus* and *P. maculosus”*

L.529: A recent study on ITS1 sequence divergence and length (Chow et al. 2023, Crust. Res. 52) showed well separate status between Pagurus and Lithodid. No discussion for this?

We appreciate the reviewer for calling our attention to this paper. Interestingly, the paper showed that lithodoid species had unique internal transcribed spacer 1 (ITS1), which evolved from those found among paguroid species. Their results support the evolution of monophyletic Lithodid from Pagurus lineage. In other words, their findings support the “hermit-to-king” crab hypothesis, as our findings do. Please see Fig. 8 of Chow *et al.* (2023) which presents the hypothetical evolutionary relationship among hermit crab families. We have included the reference in our manuscript.

Again, we thank the reviewer for their contribution. We hope all the concerns of the reviewer has been addressed.

Reviewer #2: The MS investigates the molecular and gene expression evolution of two closely related Pagurus species using transcriptome sequencing. The results sheds light on the evolutionary history of the two species at the molecular level. This MS demonstrates substantive content, sound data analysis, and reliable results, presented through well-structured writing that meets the criteria for publication readiness.

We appreciate the reviewer for the positive feedback about our manuscript. Thank you.
